# Angiopoietin-like protein 2 induces androgen-independent and malignant behavior in human prostate cancer cells

**DOI:** 10.3892/or.2014.3586

**Published:** 2014-11-03

**Authors:** RYUTA SATO, MUTSUSHI YAMASAKI, KENICHI HIRAI, TAKANORI MATSUBARA, TAKEO NOMURA, FUMINORI SATO, HIROMITSU MIMATA

**Affiliations:** Department of Urology, Oita University Faculty of Medicine, Hasama, Yufu-city, Oita 879-5593, Japan

**Keywords:** ANGPTL2, prostate cancer, EMT, apoptosis, integrin α5β1

## Abstract

Angiopoietin-like proteins (ANGPTLs), which comprise 7 members (ANGPTL1-ANGPTL7), structurally resemble angiopoietins. We investigated the roles of ANGPTLs in the acquisition of androgen independence and the malignant behavior of human prostate cancer cells. Expression of ANGPTL messenger RNA (mRNA) and proteins were ascertained using RT-qPCR and western blot analysis in human prostate cancer cell lines. Androgen-dependent LNCaP and androgen-independent LNCaP/AI cells, respectively, were cultured in fetal bovine and charcoal-stripped medium. Cell proliferation, androgen dependence, migration and invasion, respectively, were examined under the overexpression and knockdown of ANGPTL2 by transfection of ANGPTL2 cDNA and its small-interfering RNA (siRNA). The effects of exogenous ANGPTL2 and blocking of its receptor, integrin α5β1, were also investigated. Human prostate cancer cell lines predominantly expressed ANGPTL2 among the members. Interrupting ANGPTL2 expression with siRNA suppressed the proliferation, migration and invasion of LNCaP cells. LNCaP/AI cells showed a higher ANGPTL2 expression than that of LNCaP cells. Furthermore, siRNA led to apoptosis of LNCaP/AI cells. The ANGPTL2-overexpressing LNCaP cells markedly increased proliferation, epithelial-to-mesenchymal transition (EMT) and malignant behavior in androgen-deprived medium. The migration rates were increased depending on the concentration of ANGPTL2 recombinant protein and were inhibited by anti-integrin α5β1 antibodies. To the best of our knowledge, this is the first study to elucidate the expression of ANGPTL2 in human prostate cancer cells. ANGPTL2 may be important in the acquisition of androgen independency and tumor progression of prostate cancer in an autocrine and/or paracrine manner via the integrin α5β1 receptor. Targeting ANGPTL2 may therefore be an efficacious therapeutic modality for prostate cancer.

## Introduction

In developed countries, prostate cancer is the second most frequently diagnosed cancer and the third most common cause of mortality from cancer in males. Androgen ablation therapy is crucial in the treatment of patients with advanced prostate cancer. However, most patients relapse within 2–3 years after the treatment. This treatment-resistant prostate cancer, known as androgen-independent or castration-resistant prostate cancer, is the final stage of this disease (1–3). Therefore, identification of the molecules involved and novel therapeutic strategies targeting these molecules in advanced prostate cancer are required.

Seven angiopoietin-like proteins (ANGPTLs) have been identified. These ANGPTLs structurally resemble angiopoietins: they have a coiled-coil domain at the N-terminus and a fibrinogen-like domain at the C-terminus. However, ANGPTLs do not bind to the angiopoietin-specific receptor Tie2 (4–11). Recently, Aoi *et al* reported that ANGPTL2 increases inflammatory carcinogenesis in chemically induced skin squamous cell carcinoma (12). Additionally, Endo *et al* reported that ANGPTL2 expression in lung tumor cells is highly correlated with the frequency of tumor cell metastasis (13). Integrin α5β1, which acts as functional receptor for ANGPTL2 in endothelial cells and monocytes/macrophages (14,15), is also expressed in several cancer cells, in which it regulates tumor cell growth and invasion (16,17). ANGPTL2 is expressed in certain tumor cell types (18). Cancer cell-derived ANGPTL2 is an important factor in cancer development.

This study investigated the possible expression and role of ANGPTLs in human prostate cancer cells. To the best of our knowledge, this is the first study to demonstrate that a high ANGPTL2 expression induces androgen-independent and malignant behavior in human prostate cancer cells. By contrast, decreasing ANGPTL2 levels in human prostate cancer cells attenuated cell growth and malignant behavior. Our findings suggest that blocking ANGPTL2 is useful as a therapeutic strategy against prostate cancer progression.

## Materials and methods

### Cell line and culture conditions

The LNCaP, PC-3, DU145 and 22Rv1 human prostate cancer cell lines were purchased from the American Type Culture Collection (ATCC; Rockville, MD, USA). These cells were cultured at 37°C in a humidified incubator containing 5% CO_2_ and 95% air. LNCaP, DU145 and 22Rv1 cells were cultured in RPMI-1640 (Sigma-Aldrich Corp. St. Louis, MO, USA) supplemented with 15% fetal bovine serum (Sigma-Aldrich Corp.), 50 μg/ml streptomycin and 50 IU/ml penicillin (Gibco, Grand Island, NY, USA). PC-3 cells were cultured in RPMI-1640 supplemented with 10% newborn calf serum (Equitech-Bio Inc., Kerrville, TX, USA), 50 μg/ml streptomycin and 50 IU/ml penicillin.

For androgen [dihydrotestosterone (DHT)] ablation, an androgen-independent prostate cancer cell line model LNCaP/AI was cultured in phenol red free RPMI-1640 (Sigma-Aldrich Corp.) supplemented with 15% charcoal/dextran-treated fetal bovine serum (HyClone, Logan, UT, USA), 50 μg/ml streptomycin and 50 IU/ml penicillin for 3 months.

### RNA isolation and quantitative reverse-transcription polymerase chain reaction (RT-qPCR)

Total Ribonucleic acid (RNA) was isolated using TRIzol reagent (Invitrogen Life Technologies, Carlsbad, CA, USA) according to the manufacturer’s instructions. Total RNA (1 μg) was synthesized into cDNA using the ThermoScript RT-PCR System (Invitrogen Life Technologies) according to the manufacturer’s instructions. After the reverse transcription reaction, first-strand cDNA (2 μg) was used for PCR with a LightCycler^®^ FastStart DNA Master SYBR-Green I reaction mix (Roche Molecular Biochemicals, Mannheim, Germany) and QuantiTect Primer Assays (Qiagen Inc., Hilden, Germany) on a LightCycler system (Roche Diagnostics Corp., Indianapolis, IN, USA). Each cycle included denaturation at 95°C for 15 sec, annealing at 55°C for 5 sec and polymerization at 72°C for 10 sec. The primers used were ANGPTL2 (HS_ANGPTL2_1_SG QuantiTect Primer Assay; QT00091021) and β-actin (HS_ACTB_1_SG QuantiTect Primer Assay; QT00095431). Messenger RNA (mRNA) expression quantification was normalized by β-actin mRNA expression.

### Immunohistochemical staining

Cultured cells were washed with phosphate-buffered saline (PBS), fixed in methanol for 20 min and incubated in 10% goat normal serum (Nichirei Corp., Tokyo, Japan) for 10 min at 37°C. The cells were incubated in the primary antibodies against ANGPTL2 at a dilution of 1:500 (Atlas Antibodies AB, Stockholm, Sweden), integrin α5β1 at a dilution of 1:500 (Biorbyt Ltd., Cambridge, UK) at room temperature in PBS with 1% BSA for 60 min. After incubation with primary antibodies, the secondary antibody (anti-rabbit; Dako, Kyoto, Japan) was added to PBS with 1% BSA for 30 min. After washing with PBS, the color was developed using the Dako Cytomation Liquid DAB Substrate Chromogen System (Dako). The cells were counterstained with hematoxylin.

Prostate cancer specimens were obtained from prostate cancer patients (n=10) undergoing radical prostatectomy, with (n=5) and without (n=5) androgen ablation therapy. The study was approved by the Oita University Ethics Committee and informed consent was obtained for experimentation with human subjects. Tissue sections were cut from paraffin-embedded blocks and were placed on silicone-coated slides. After deparaffinization in xylene and rehydration using graded alcohol solutions, the sections were incubated in 0.3% H_2_O_2_ for 10 min to inactivate endogenous peroxidase with subsequent washing with PBS. To block non-specific binding to sections and to eliminate non-specific staining, 10% normal goat serum in PBS was applied to the sections and incubated for 10 min. The slides were then incubated in the primary antibody against ANGPTL2 at a dilution of 1:250 (Atlas Antibodies AB) at room temperature in PBS with 1% BSA for 60 min. After incubation with primary antibodies, the secondary antibody (anti-rabbit; Dako) was added to PBS with 1% BSA for 30 min. After washing with PBS, the color was developed using the Dako Cytomation Liquid DAB Substrate Chromogen System (Dako). The cells were counterstained with hematoxylin.

### Transient transfection of ANGPTL2 small-interfering RNA (siRNA)

LNCaP and LNCaP/AI cells were transiently transfected with a ANGPTL2 siRNA duplex [si-ANGPTL2; final concentration, 60 nmol/l (Qiagen Inc.)] or control siRNA [random scrambled sequence: si-Scr; final concentration, 60 nmol/l (Qiagen Inc.)] using Lipofectamine RNAiMAX (Invitrogen Life Technologies) according to the manufacturer’s instructions. The sequence of the siRNA against ANGPTL2 generated by Invitrogen was: 5′-AACCTGAGAGCGAGTATTATA(dT)(dT)-3′, 5′-CTCGCGGGTCACGCAGCTCTA(dT)(dT)-3′, 5′-ACCGGCCGTATAGATAATGTA(dT)(dT)-3′, 5′-CAGAATGTCTACAATGCTAAT(dT)(dT)-3′.

### Cell proliferation assays

The cells were seeded in 24-well plates at a density of 5×10^4^ cells/well. The cells were trypsinized, collected and counted at 24, 48 and 72 h using hemocytometry.

### Migration and invasion assays

Cell migration was assessed using a 24-well BioCoat Control Insert Chamber (BD Biosciences, Franklin Lakes, NJ, USA) with polycarbonate filters containing 8-μm pores. The cells were plated at 5×10^4^ cells/well in 0.5 ml of serum-free medium. The outer chambers were filled with 0.75 ml of media containing 15% fetal bovine serum. After 48 h, cells migrating to the undersurface of the filters were counted. The top surface of the membrane was scrubbed gently with a cotton swab. The cells on the undersurface were then fixed in 99.8% methanol and stained with 0.05% toluidine blue prior to undergoing a series of washes. The cells passing to the undersurface of each filter were counted using a cell counter system (BZ-9000; Keyence Co., Osaka, Japan).

For invasion assays, the control insert chambers were replaced with BioCoat Matrigel Invasion Chambers (BD Biosciences) treated with a Matrigel Matrix reconstituted basement membrane layer.

For migration inhibition analysis by integrin α5β1 immunoneutralization, the outer chambers were filled with 0.75 ml of media containing ANGPTL2 recombinant protein (Adipogen Corp. San Diego, CA, USA) of various concentrations (0, 1.0, 2.5 and 5.0 μg/ml) with 25 μg/ml mouse anti-human integrin α5β1 monoclonal antibody (Millipore Corp. Billerica, MA, USA) or 25 μg/ml control mouse IgG (Zymed Laboratories Inc., San Francisco, CA, USA).

### Protein extraction and western blot analysis

Proteins were extracted from cell plates with lysis buffer [50 mmol/l Tris (pH 8.0), 150 mmol/l NaCl, 0.02% NaN_3_, 0.1% sodium dodecyl sulfate, 1% NP-40, 0.5% sodium deoxycholate and 1 mmol/l phenylmethylsulfonyl fluoride] in the presence of a protease inhibitor cocktail (Roche Applied Science, Indianapolis, IN, USA). Samples containing equal amounts of protein (20 μg) were electrophoresed on 4–20% Tris-glycine gels (Tefco Corp., Tokyo, Japan) and transferred to nitrocellulose membranes. After blocking with Blocking One-P solution (Nacalai Tesque Inc., Kyoto, Japan), the membranes were incubated with mouse monoclonal antibodies against β-tubulin at a dilution of 1:5,000 (Sigma-Aldrich Corp.) or rabbit polyclonal antibodies against ANGPTL2 at a dilution of 1:1,000 (Proteintech Group, Chicago, IL, USA), Caspase-9, Caspase-3, poly adenosine diphosphate-ribose polymerase (PARP), Bcl-2 at a dilution of 1:1,000 (Santa Cruz Biotechnology, Inc., Santa Cruz, CA, USA), Bcl-xL, Bad, E-Cadherin, N-Cadherin, vimentin, snail, slug at a dilution of 1:1,000 (Cell Signaling Technology, Inc., Danvers, MA, USA) and integrin α5β1 at a dilution of 1:1,000 (Biorbyt Ltd.) at 4°C overnight. After washing with Tween-Tris-buffered saline (T-TBS), the membranes were incubated with the corresponding secondary antibodies conjugated with horseradish peroxidase in T-TBS for 1 h at room temperature. Immunoreactive bands were visualized using a western blotting detection system (ECL Plus; Amersham Pharmacia Biotech, Little Chalfont, UK).

### Stable ANGPTL2 transfection

LNCaP cells were transfected with ANGPTL2 expression vector (OriGene Technologies Inc. Rockville, MD, USA) or empty control vector (Neo; OriGene Technologies Inc.) using Optifect reagent (Invitrogen Life Technologies) according to the manufacturer’s instructions. Briefly, LNCaP cells were seeded in six-well plates at a density of 2×10^5^ per well 24 h prior to transfection in an FBS-supplemented medium. The cells were transfected using 4 μg of ANGPTL2 expression vector or Neo and 18 μg Optifect reagent/well and cultured in the presence of 500 μg/ml geneticin sulfate to obtain stable transfectants.

### Statistical analysis

Values were presented as means ± SD. Statistical analyses were performed using the Student’s t-test. P<0.05 were considered statistically significant.

## Results

### Expression of ANGPTLs in prostate cancer cell lines

**Figure 1:**
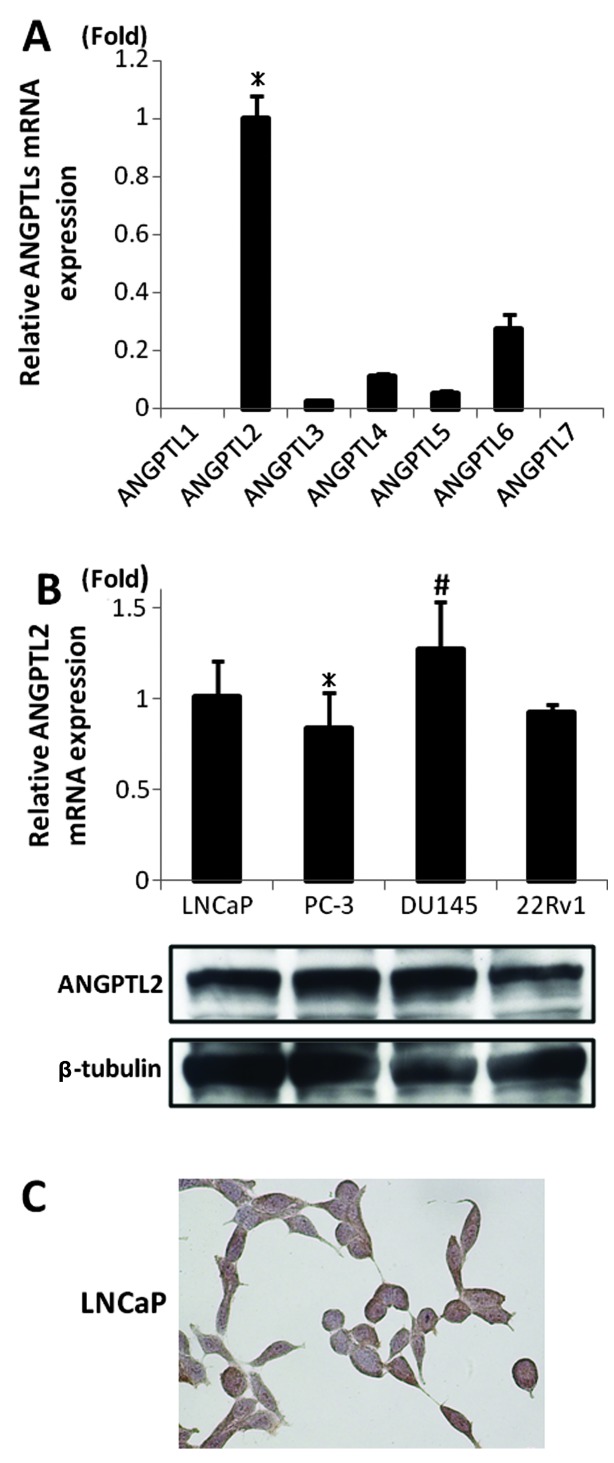
Expression of angiopoietin-like proteins (ANGPTLs) messenger RNA (mRNA) and protein. (A) Expression of ANGPTL families in LNCaP cells was analyzed using quantitative reverse-transcription polymerase chain reaction (RT-qPCR). The expression of ANGPTL2 mRNA was significantly increased (^*^p<0.05 vs. ANGPTL6). (B) ANGPTL2 mRNA and protein expression was confirmed using RT-qPCR and western blot analysis in LNCaP, PC-3, DU 145 and 22Rv1 cells (^*^p=0.34, ^#^p=0.20 vs. LNCaP). (C) Immunohistochemical staining of LNCaP cells. β-tubulin served as the control.

The expression of ANGPTL families in LNCaP cells was analyzed using RT-PCR. The expression of ANGPTL2 mRNA was increased significantly (p<0.05; Fig. 1A). The expression of mRNA and protein of ANGPTL2 was confirmed in the other prostate cancer cell lines (PC-3, DU145 and 22Rv1) using western blot analysis and immunohistochemical staining (Fig. 1B and C).

**Figure 2:**
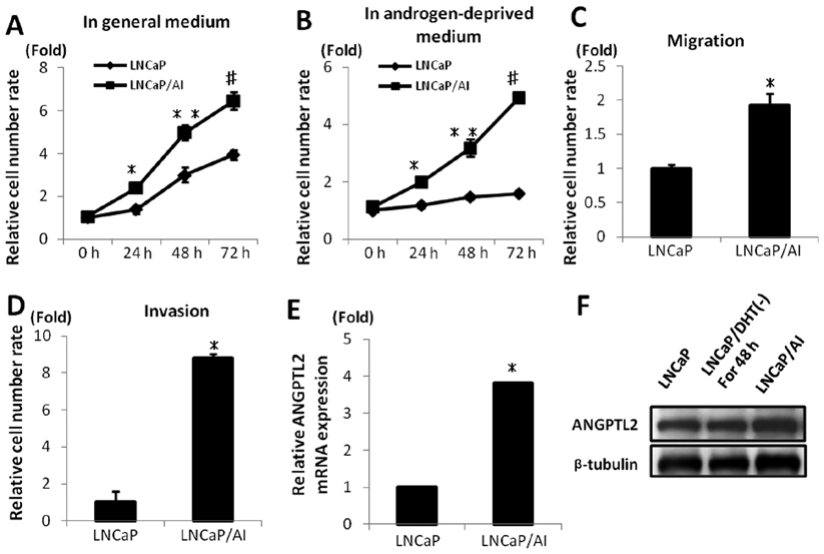
Angiopoietin-like proteins (ANGPTL2) expression levels in LNCaP/AI cells. (A–D) LNCaP/AI cells showed significantly increased cell proliferation, migration and invasion compared with LNCaP cells (A, ^*^p<0.05, ^**^p<0.03, ^#^p=0.02, B, ^*^p<0.05, ^**^p<0.01, ^#^p<0.001, C, D, ^*^p<0.05). (E and F) Quantitative reverse transcription polymerase chain reaction (RT-qPCR) and western blot analysis revealed that ANGPTL2 was significantly more expressed in LNCaP/AI cells than in LNCaP cells (^*^p<0.05). β-tubulin.served as the control.

An androgen-independent prostate cancer cell line model, LNCaP/AI, showed increased cell proliferation, migration and invasion than the androgen-dependent prostate cancer cell line, LNCaP (p=0.02; Fig. 2A, p<0.001; Fig. 2B, p<0.05; Fig. 2C and D). RT-PCR and western blot analysis revealed that ANGPTL2 mRNA and protein were expressed at higher levels in LNCaP/AI cells than those in LNCaP cells (p<0.05; Fig. 2E and F).

### Effects of ANGPTL2 siRNA on cell proliferation, migration, invasion, apoptosis of LNCaP and LNCaP/AI cells

**Figure 3:**
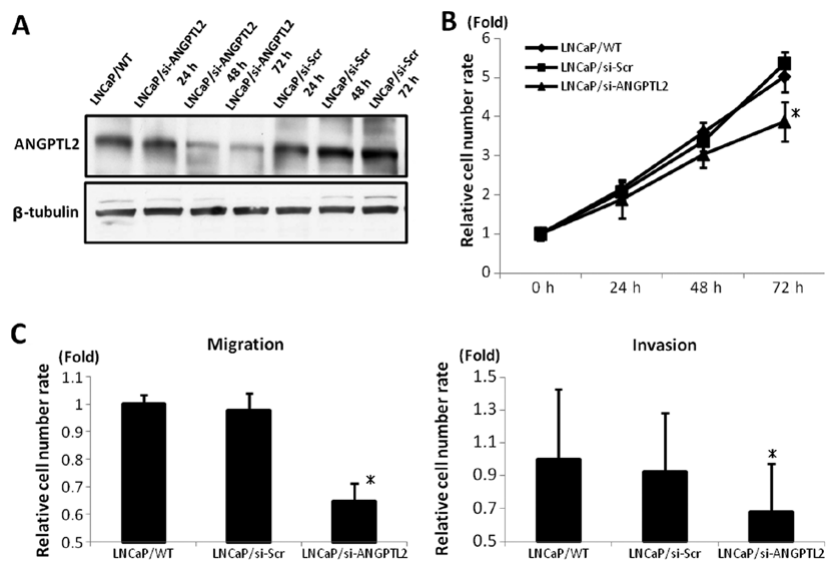
Effects of angiopoietin-like proteins (ANGPTL2) small-interfering RNA (siRNA) on cell proliferation, migration and invasion in LNCaP cells. (A) ANGPTL2 siRNA downregulated ANGPTL2 expression for 72 h after transfection into LNCaP cells. (B) LNCaP/si-ANGPTL2 cell growth was more restricted than in LNCaP/wild-type (WT) or LNCaP/si-Scr (^*^p<0.05). (C) LNCaP/si-ANGPTL2 reduced cell migration and invasion significantly more than in LNCaP/WT or LNCaP/si-Scr (^*^p<0.05).

ANGPTL2 siRNA reduced the expression of ANGPTL2 protein in LNCaP cells from 24 to 72 h after transfection (Fig. 3A). The proliferation assay revealed that the cell growth of LNCaP/si-ANGPTL2 was more suppressed than that of LNCaP/wild-type (WT) or LNCaP/si-Scr (p<0.05; Fig. 3B).

Cell migration and invasion of LNCaP/si-ANGPTL2 were reduced compared with those of LNCaP/WT and LNCaP/si-Scr (p<0.05; Fig. 3C).

These results suggested that ANGPTL2 is involved in the proliferation, migration and invasion of LNCaP cells and that ANGPTL2 siRNA negatively affected these behaviors.

**Figure 4:**
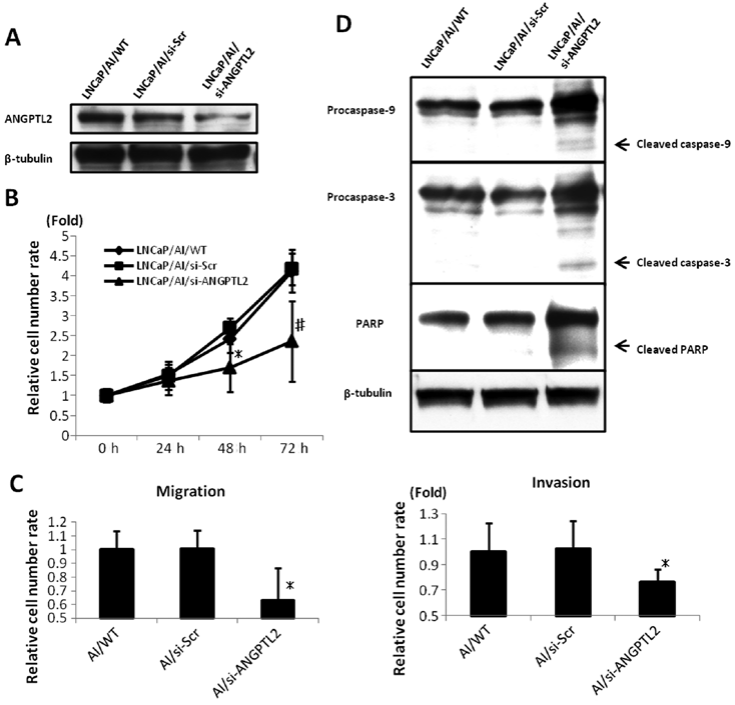
Effects of angiopoietin-like proteins (ANGPTL2) small interfering RNA (siRNA) on cell proliferation, migration and invasion in LNCaP/AI cells. (A) ANGPTL2 siRNA downregulated ANGPTL2 expression after transfection into LNCaP/AI cells. (B) LNCaP/AI/si-ANGPTL2 cell growth was significantly more restricted than that in LNCaP/AI/WT or LNCaP/AI/si-Scr (^*^p<0.05, ^#^p<0.02). (C) LNCaP/AI/si-ANGPTL2 more significantly reduced cell migration and invasion than that in LNCaP/AI/WT or LNCaP/AI/si-Scr (^*^p<0.05). (D) Activation of caspase-9 and caspase-3 and poly adenosine diphosphate-ribose (PARP) cleavage after transfection with ANGPTL2 siRNA or si-Scr were determined using western blot analysis. Treatment with ANGPTL2 siRNA induced caspase-9 and caspase-3 activation and PARP cleavage in LNCaP/AI cells.

ANGPTL2 siRNA downregulated the ANGPTL2 protein expression 72 h after transfection in LNCaP/AI cells (Fig. 4A). The LNCaP/AI/si-ANGPTL2 cell growth was more restricted than that of LNCaP/AI/WT or LNCaP/AI/si-Scr (p<0.02; Fig. 4B).

Cell migration and invasion were also assessed. LNCaP/AI/si-ANGPTL2 reduced cell migration and invasion more than LNCaP/AI/WT or LNCaP/AI/si-Scr (p<0.05; Fig. 4C).

To investigate whether ANGPTL2 siRNA-induced growth inhibition was triggered by increased apoptosis, we investigated caspase-9 and caspase-3 activation and subsequent PARP cleavage using western blot analysis. Treatment with ANGPTL2 siRNA induced caspase-9 and caspase-3 activation and PARP cleavage in LNCaP/AI cells (Fig. 4D).

These results suggested that ANGPTL2 induced the malignant potential in androgen-dependent prostate cancer cell line LNCaP cells and in androgen-independent prostate cancer cell line model LNCaP/AI cells.

### Effects of ANGPTL2 overexpression on cell proliferation, migration, invasion and acquisition of androgen independence in LNCaP cells

**Figure 5:**
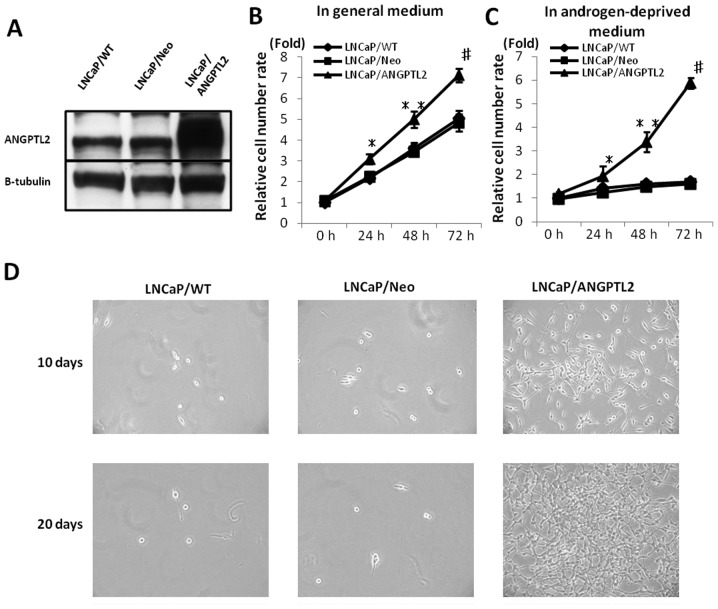
Effects of angiopoietin-like proteins (ANGPTL2) overexpression on cell proliferation, migration and invasion in LNCaP cells. (A) Human ANGPTL2 cDNA expression vector upregulated ANGPTL2 expression following transfection into LNCaP cells. (B), (C) ANGPTL2-overexpressing transfectant growth rates were significantly higher than those of LNCaP/WT or LNCaP/Neo in the general and androgen-deprived media (B, ^*^p<0.02, ^**^p<0.02, ^#^p<0.01, C, ^*^p<0.01, ^**^p<0.01, ^#^p<1.0E-07). (D) LNCaP/ANGPTL2 has upregulated cell growth for 10 and 20 days in androgen-deprived medium.

The results showed that LNCaP cells were stably transfected with the human ANGPTL2 cDNA expression vector. Clonal ANGPTL2-overexpressing LNCaP cell lines were established. LNCaP stably transfected with Neo serving as a control. The human ANGPTL2 cDNA expression vector upregulated ANGPTL2 expression following transfection into LNCaP cells (Fig. 5A). The ANGPTL2-overexpressing transfectant growth rates were higher than those of LNCaP/WT or LNCaP/Neo, in the general and androgen-deprived media. The LNCaP/ANGPTL2 upregulated cell growth for 20 days longer after placement into androgen-deprived medium than LNCaP/WT or LNCaP/Neo (p<0.01; Fig. 5B, p<1.0E-07; Fig. 5C and D).

**Figure 6:**
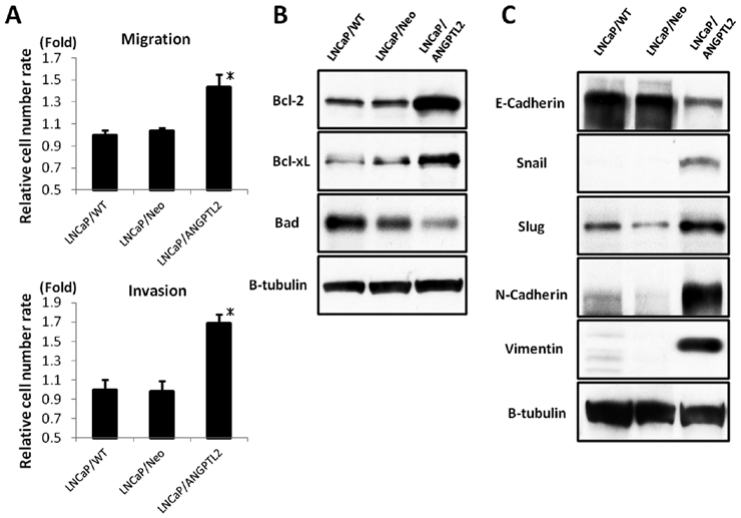
Cell migration and invasion of the angiopoietin-like proteins (ANGPTL2)-overexpressing transfectants and expressions of apoptosis and epithelial-to-mesenchymal transition (EMT)-associated proteins. (A) LNCaP/ANGPTL2 showed significantly increased cell migration and invasion than that in LNCaP/WT or LNCaP/Neo (^*^p<0.01). (B) LNCaP/ANGPTL2 increased the expression levels of Bcl-2 and Bcl-xL proteins and decreased the expression level of Bad protein. (C) LNCaP/ANGPTL2 increased the expression levels of snail, slug, N-Cadherin and vimentin proteins and decreased the expression level of E-Cadherin protein.

We also analyzed cell migration and invasion. The ANGPTL2-overexpressing transfectants increased cell migration and invasion more than that of LNCaP/WT or LNCaP/Neo (p<0.01; Fig. 6A).

Additionally, we used western blot analysis to assess expression levels of anti-apoptotic proteins Bcl-2 and Bcl-xL and pro-apoptotic protein Bad. The ANGPTL2- overexpressing transfectants increased the expression levels of Bcl-2 and Bcl-xL proteins and decreased the expression level of Bad protein more than that of LNCaP/WT or LNCaP/Neo (Fig. 6B).

A critical event in tumor metastasis is a decrease in cancer cell adhesion through acquisition of mesenchymal phenotypes and invasive properties. The epithelial-to-mesenchymal transition (EMT) correlated with ANGPTL2 expression levels. Western blot analysis revealed that the ANGPTL2-overexpressing transfectants increased the expression levels of snail, slug, N-Cadherin and vimentin proteins, as mesenchymal markers, and decreased the expression level of E-Cadherin protein, as an epithelial indicator, more than that of LNCaP/WT or LNCaP/Neo (Fig. 6C).

These results suggested that ANGPTL2 overexpression induced androgen-independent progression and malignant behavior in LNCaP cells via escape from apoptosis and promotion of EMT.

### Integrin α5β1 expression levels in LNCaP and LNCaP/AI cells

**Figure 7:**
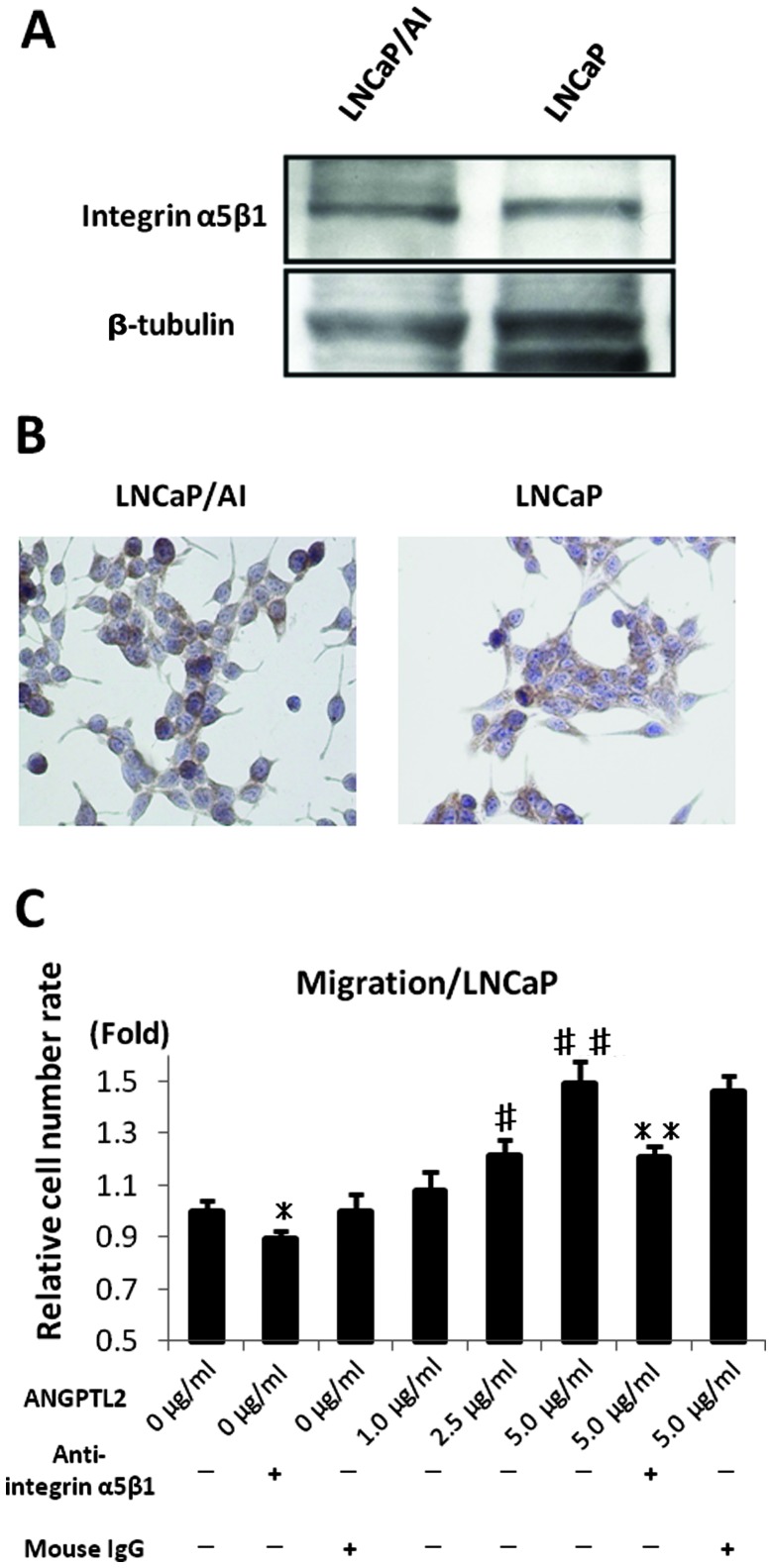
Integrin α5β1 expression levels in LNCaP and LNCaP/AI cells. Effects of integrin α5β1 immunoneutralization and angiopoietin-like (ANGPTL2) recombinant protein on cell migration in LNCaP cells. (A and B) Expression of integrin α5β1 protein in LNCaP and LNCaP/AI cells was confirmed using western blot analysis and immunohistochemical staining. (C) Cell migration with integrin α5β1 immunoneutralization was significantly more restricted than that in LNCaP/WT or LNCaP/control mouse IgG. Migration rates were increased significantly depending on the concentration of ANGPTL2 recombinant protein (^*^p<0.05 vs. anti-integrin α5β1(−). ^**^p<0.05 vs. ANGPTL2 (5 μg/ml), anti-integrin α5β1(−). ^#^p<0.05 vs. ANGPTL2 (0 μg/ml). ^##^p<0.01 vs. ANGPTL2 (0 μg/ml).

Integrin α5β1 is not specific receptor of ANGPTL2, but a functional receptor of ANGPTL2. The expression of integrin α5β1 protein in LNCaP and LNCaP/AI cells was confirmed using western blot analysis and immunohistochemical staining (Fig. 7A and B).

### Effects of integrin α5β1 immunoneutralization and ANGPTL2 recombinant protein on cell migration in LNCaP cells

To assess the effect of integrin α5β1 immunoneutralization in LNCaP cells, we analyzed cell migration. Cell migration with integrin α5β1 immunoneutralization was more restricted than in LNCaP/WT or LNCaP/control mouse IgG (p<0.05; Fig. 7C). To assess the effects of the autocrine/paracrine action of ANGPTL2, we analyzed cell migration in LNCaP cells with various concentrations (0, 1.0, 2.5 and 5.0 μg/ml) of ANGPTL2 recombinant protein. The migration rates increased along with the ANGPTL2 recombinant protein concentration (p<0.01; Fig. 7C).

These results suggested that ANGPTL2 affected human prostate cancer cells in an autocrine/paracrine manner via the integrin α5β1 receptor.

### ANGPTL2 expression levels in human prostate cancer tissues

**Figure 8:**
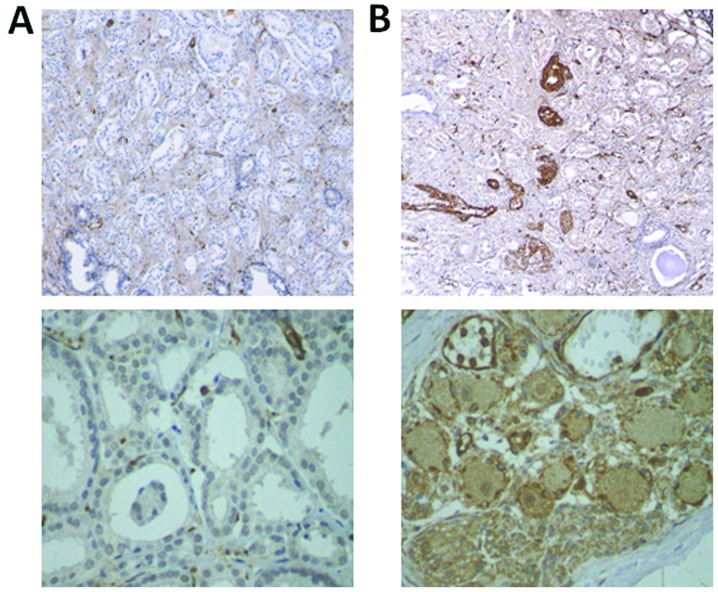
Angiopoietin-like proteins (ANGPTL2) expression levels in human prostate cancer tissues. (A) Prostate cancer tissue without androgen ablation therapy (Gleason score 3+3=6, well differentiated). (B) Prostate cancer tissue after androgen ablation therapy (Gleason score 3+4=7, well differentiated). Weakly positive staining of ANGPTL2 was observed in specimens obtained from patients without neoadjuvant hormonal therapy. However surviving prostate cancer cells were strongly positive-staining after hormonal therapy.

Immunohistochemical staining revealed that ANGPTL2 was expressed at higher levels in human prostate cancer tissues after androgen ablation therapy than in human prostate cancer tissues without androgen ablation therapy (Fig. 8A and B).

These results suggested that androgen ablation therapy induced more ANGPTL2 expression in human prostate cancer tissues.

## Discussion

Prostate cancer cells require androgens for growth and survival. Androgen ablation therapy is therefore the gold standard for advanced prostate cancer. However, androgen-dependent prostate cancer becomes androgen-independent with long-term androgen deprivation. Both androgen receptor (AR)-dependent and AR-independent mechanisms are involved in progression to the castration-resistant state (19,20). The role of ANGPTL2 in prostate cancer development has not been reported in connection with androgen independency and malignant behavior. Therefore, the aims of this study were i) to define the biological effects of ANGPTL2 in prostate cancer cells, and ii) to examine the role of ANGPTL2 as a novel therapeutic target for prostate cancer.

The results of this study demonstrate that ANGPTL2 expression is strongly induced by androgen deprivation and that it is highly associated with androgen-independent and malignant behavior in LNCaP cells in an autocrine and/or paracrine manner via the integrin α5β1 receptor. The downregulation of ANGPTL2 expression by siRNA inhibits cell growth, migration and invasion of LNCaP cells and androgen-independent prostate cancer cell line model LNCaP/AI. Furthermore, stably overexpressed ANGPTL2 promotes cancer cell growth, migration and invasion, in the general and androgen-deprived media over the long term via escape from apoptosis and promotion of EMT. Thus, ANGPTL2 is an effective growth-promoting factor and an important determinant of the potential malignancy of androgen-independent prostate cancer cells. Additionally, we have demonstrated that strong immunostaining of ANGPTL2 occurred in residual cancer cells in prostate specimens obtained from patients who had undergone neoadjuvant hormonal therapy. This finding suggests that ANGPTL2 is involved in the hormone refractory mechanism in clinical prostate cancer.

ANGPTL2 was found to increase inflammatory carcinogenesis in a chemically induced skin squamous cell carcinoma. Furthermore, ANGPTL2 expression in lung tumor cells is highly correlated with the frequency of tumor cell metastasis through increased tumor angiogenesis. It was also reported that ANGPTL2 increases EMT in cancer cells (12,13). Additionally, ANGPTL2 stimulates the nuclear translocation of nuclear factor-κB (NF-κB) via integrin α5β1 signaling (14). The NF-κB-dependent expression of genes is extremely important for anti-apoptotic mechanisms in cancer cells. Therefore, the activation of NF-κB induces high rates of cancer cell proliferation via the producton of anti-apoptotic proteins. The abovementioned studies supported the findings of this study.

Kikuchi *et al* reported ANGPTL2 as a putative tumor suppressor in ovarian cancer (18). In that study, lack of ANGPTL2 immunoreactivity was associated with poorer overall survival in stage I and II disease, whereas ANGPTL2 positivity was associated significantly with a poorer survival in stage III and IV disease. The latter findings are consistent with those of this study, suggesting that ANGPTL2 is critical for tumor progression and malignant behavior. However, we did not observe a tumor suppressor function for ANGPTL2 in human prostate cancer cells. Whether ANGPTL2 function differs in various tumor cell types remains to be determined (21–23). Additionally, ANGPTL1 represses lung cancer cell motility by abrogating the expression of the EMT mediator slug (24). However, it has been reported that ANGPTL4 promotes cancer growth and progression (25–27). The involvement of the ANGPTLs family in cancer remains unclear and requires further investigation.

In conclusion, ANGPTL2, which is upregulated in androgen-independent prostate cancer cells and human prostate cancer tissue after androgen ablation therapy, may be important in androgen-independent prostate cancer progression in an autocrine and/or paracrine manner via the integrin α5β1 receptor. Downregulation of ANGPTL2 by siRNA reduces migration and invasion and inhibits cancer cell growth. Targeting ANGPTL2 may therefore be an efficacious therapeutic modality for prostate cancer, especially androgen-independent prostate cancer.
